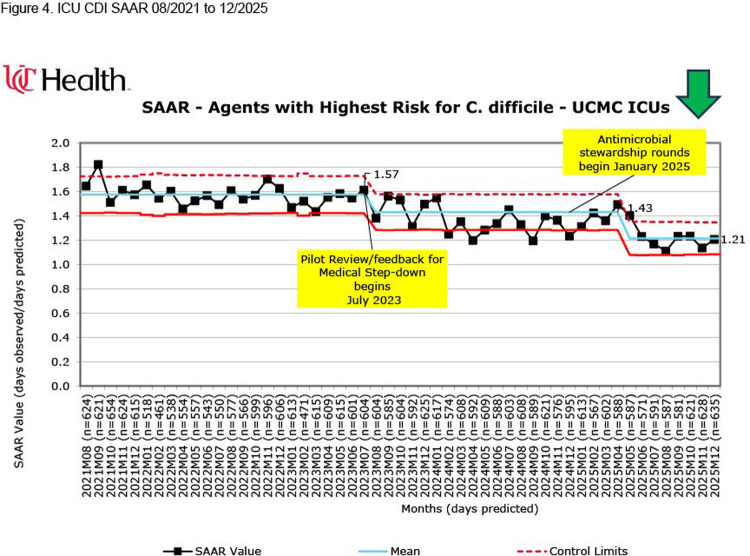# 116 Clinician Perceptions of a Novel Metric to Compare VHA Hospitals on Antibiotic Prescribing at Hospital Discharge

**DOI:** 10.1017/ash.2026.10531

**Published:** 2026-06-23

**Authors:** Kelli Williams, Tara Harpenau, Anna Poston-Blahnik, Christen Arena, Mary Palmer Klawon, Jennifer Forrester

**Affiliations:** 1 University of Cincinnati College of Medicine; 2 UC Health; 3 University of Cincinnati Medical Center - UC Health; 4 Henry Ford Hospital, Wayne State University; 5 University of Cincinnati/ UC Health

## Abstract

Clostridioides difficile (C. difficile) negatively impacts patient care and health care costs. Monitored as a safety metric, hospital onset- C. difficile infections (HO-CDI) occur after day 3 of admission. Standardized Antimicrobial Administration Ratio values for antibacterial agents posing highest risk for C. difficile infection (CDI SAAR) provides national benchmarking and assesses the impact of interventions aimed at improving prescribing practices to reduce C. difficile rates. We implemented several antimicrobial stewardship interventions to evaluate whether these reduced CDI SAAR values and C. difficile rates. Before 2023, C. difficile testing consisted of PCR alone after meeting certain criteria. This changed so that a positive PCR test reflexes to an EIA for toxin B; if negative, CDI is ruled out. Stewardship interventions in 06/2023 provided targeted education and feedback on CDI SAARs to ICU pharmacists to improve antimicrobial use. In 10/2023, a second intervention targeted high SAARs in the medical step-down unit (MSD) through daily patient review for antibiotic optimization. By early 2025, the antimicrobial stewardship pharmacist and physician initiated twice-weekly meetings to assess antimicrobial appropriateness. Patients were identified via chart review or pharmacist referral, and recommendations were communicated to the providers and documented in the chart. HO-CDI decreased both in the ICU and hospital wide after changing to two-step testing, decreasing from 0.55 to 0.25 infections/10,000 patient days (PD) hospital wide (Figure 1) and from 1 to 0.5/10,000PD in ICUs (Figure 3). In 2023, prospective audit and feedback by stewardship pharmacists in MSD patients resulted in a decrease in CDI SAAR from 1.26 to 1.12 (Figure 2). In October 2023, targeted education to ICU pharmacists coincided with reduction of ICU CDI SAAR from 1.57 to 1.43 (Figure 4). After biweekly stewardship rounds were implemented in 2025, HO-CDI decreased from 0.25 to 0.12/10,000PD hospital wide (Figure 1) and from 0.5 to 0.12/10,000PD in our ICUs (Figure 3). Special cause with 5 points at or below the line is noted in our hospital wide SAAR (Figure 2). Also noted special cause with increase in our hospital wide HO-CDI in November 2025 but an investigation did not identify a known cause. Interventions that reduce CDI SAAR values decreased HO-C difficile infections. Targeted interventions like prospective audit and feedback and educating team pharmacists on SAARs improved antibiotic utilization. Handshake stewardship rounding not only reduced SAAR but also decreased HO-CDI in our experience. Antibiotic Stewardship Programs help hospitals improve clinical outcomes and minimize harm by improving antibiotic prescribing.

**Figure:**
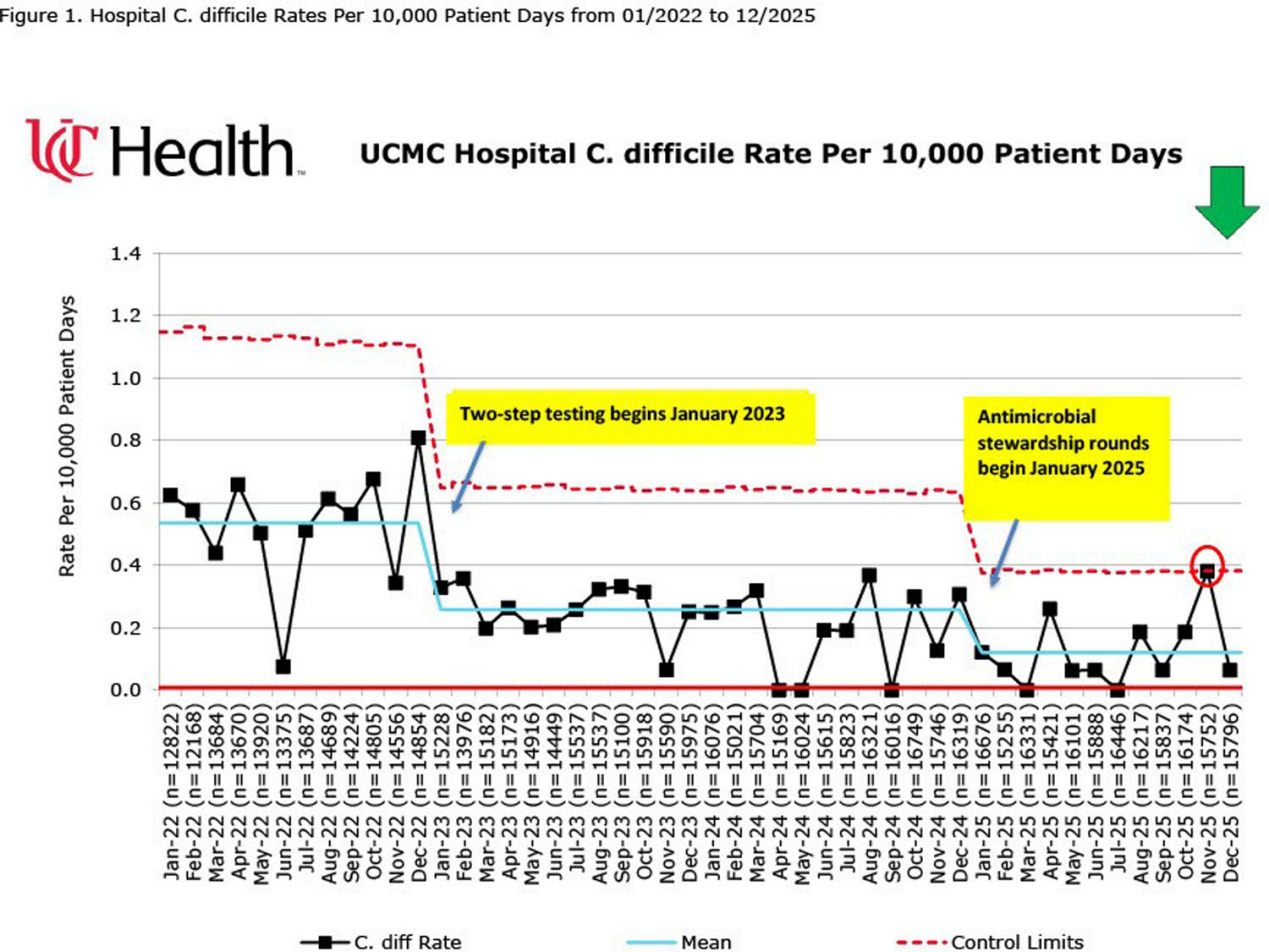

**Figure:**
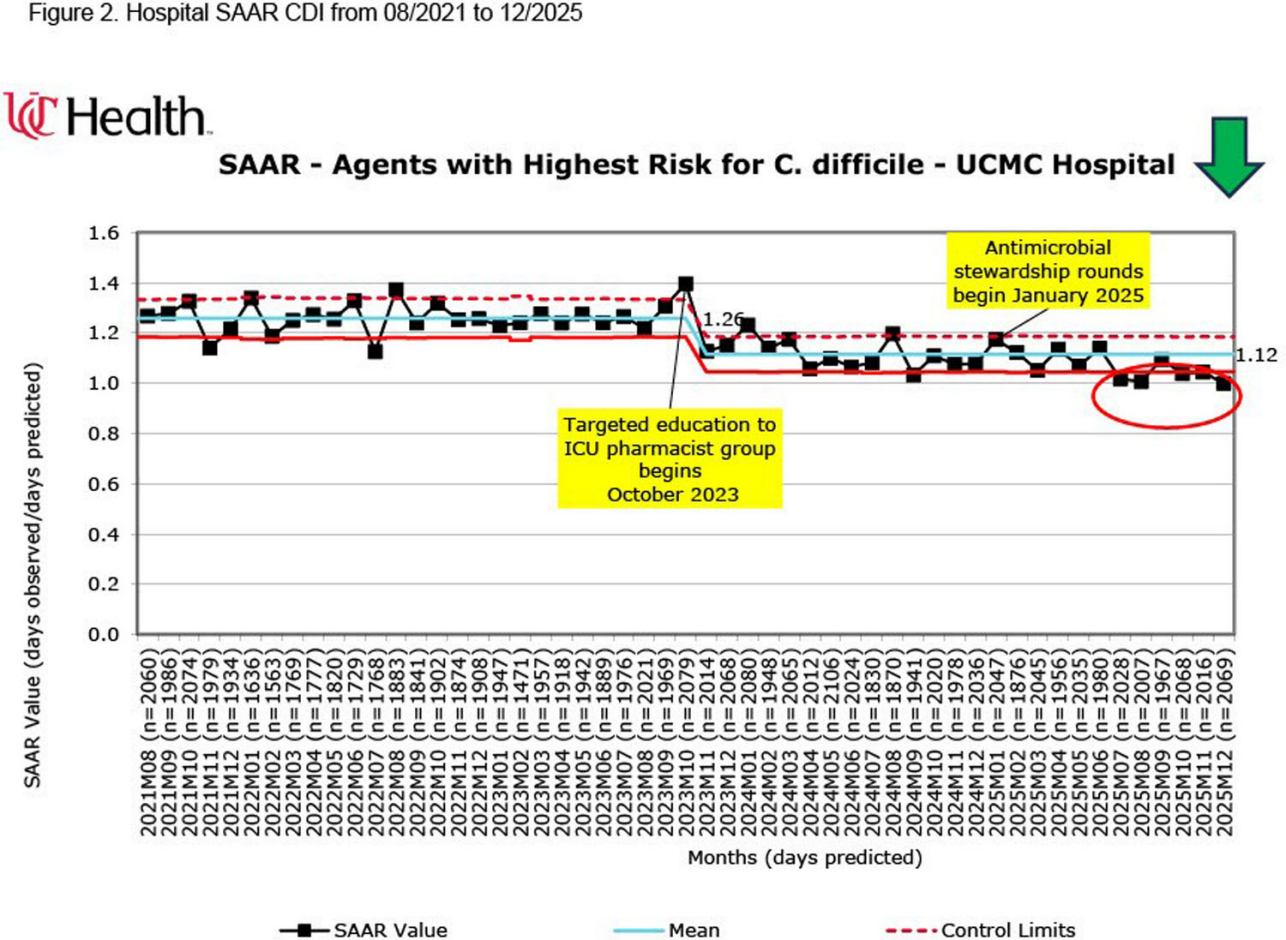

**Figure:**
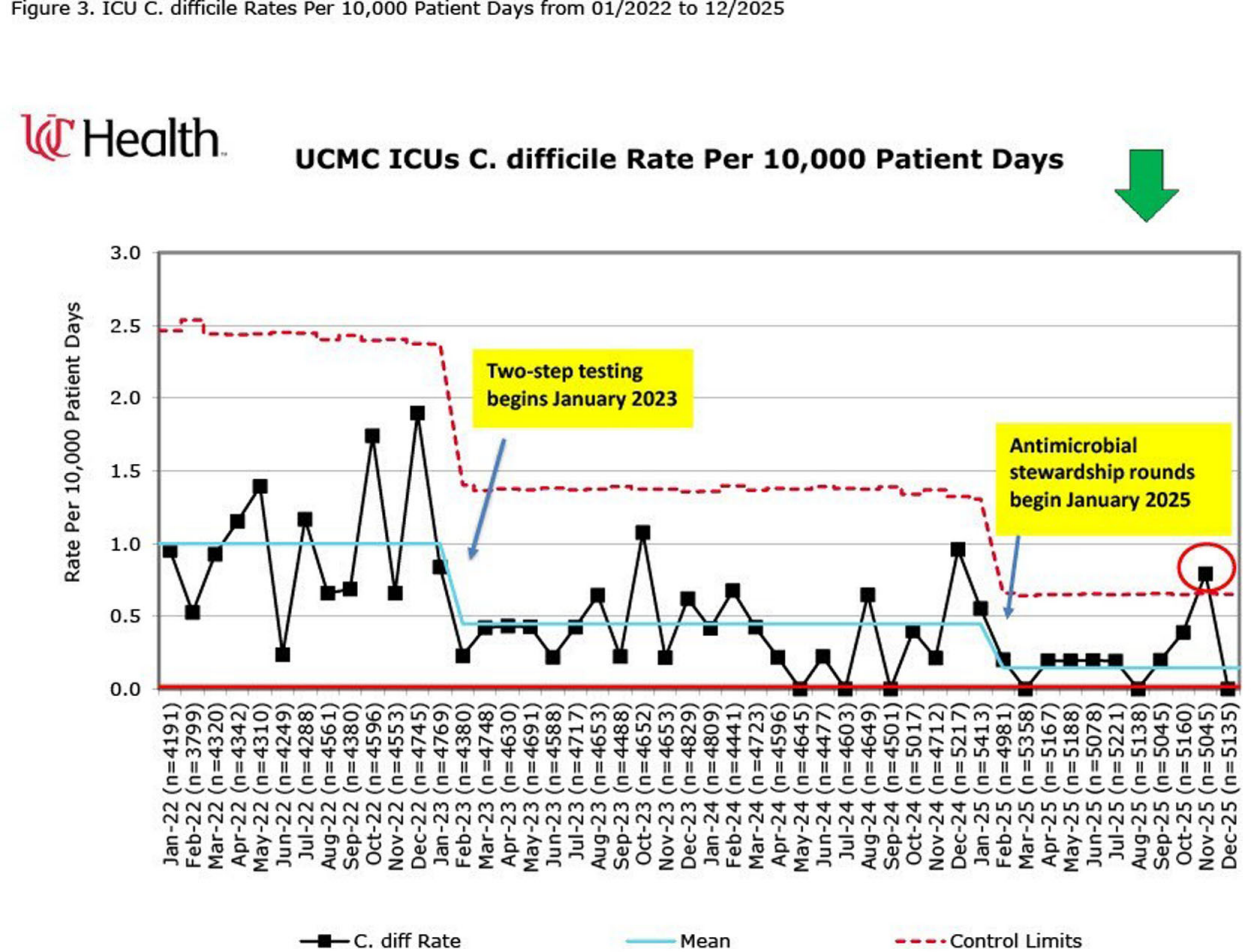

**Figure:**